# A narrative review of the effectiveness of pain management strategies during intrauterine device insertion and endometrial biopsies

**DOI:** 10.1177/17455057261442100

**Published:** 2026-04-24

**Authors:** Nora Shero, Manon Denis-LeBlanc

**Affiliations:** 1 Department of Family Medicine, Montfort Hospital, University of Ottawa, ON, Canada

**Keywords:** pain management, intrauterine devices, endometrial biopsy, patient anxiety, patient satisfaction

## Abstract

A variety of pain management techniques are currently used during gynecological procedures in outpatient settings to help improve patient comfort and satisfaction. The most common pharmacological methods include nonsteroidal anti-inflammatory drugs, lidocaine gel, lidocaine spray, and paracervical blocks. Moreover, noninvasive techniques such as transcutaneous electrical nerve stimulation and verbal analgesia have been shown to help reduce pain during intrauterine device (IUD) insertions and endometrial biopsies (EMB). More recently, Penthrox, an inhaled analgesic, is another promising new option for pain control in primary care practice. Despite the variety of available techniques, there are limited recent data evaluating the comparative efficacy of these pain management strategies during IUD insertions and EMB. Several factors play a significant role in pain perception. For example, parous women tend to have a higher tolerance for pain and report lower pain scores than nulliparous women who have not given birth and are more likely to experience cervical stenosis. This gap in evidence highlights the need for ongoing research and the development of accessible, evidence-based interventions in primary healthcare settings to enhance patient experiences and satisfaction with these common procedures. Patient education about the procedure, its benefits, and potential side effects, and providing additional counseling can help alleviate anxiety and contribute to better overall outcomes, and in turn, reduce pain.

## Introduction

Between 2017 and 2019, approximately 65% of US women aged 15–49 were using a contraceptive method.^1^ This use varies with age, from 38.7% among women aged 15–19 to 74.8% among those aged 40–49. Both the American College of Obstetricians and Gynecologists and The Society of Obstetricians and Gynaecologists of Canada (SOGC) strongly recommend intrauterine devices (IUDs) as a first-line contraceptive, including for female adolescents and nulliparous women, due to their high efficacy and safety.^2,3^ However, the fear of pain during insertion is a major obstacle to their use.^4^ This is because specific steps in the procedure can be particularly painful, including the placement of the tenaculum, the uterine catheter, the cervical crossing, and contact with the fundus. Nulliparous status is often associated with greater pain.^5^ These fears can dissuade many women, especially the youngest, from choosing this contraceptive method, which is nevertheless advantageous. Up to 30% of women will seek medical assistance due to an abnormal uterine bleed and must undergo endometrial sampling if they have any risk of endometrial hyperplasia and cancer.^6^

Several techniques exist to minimize pain during gynecological procedures such as IUD insertion or endometrial biopsies (EMB). Both procedures are common outpatient procedures that involve uterine instrumentation and utilize similar cervical access techniques, which can result in comparable pain in patients.^7^ They also share similar procedural risks such as perforation, infection, and vasovagal reaction. Thus, health professionals will use similar pain management strategies such as analgesia, local anesthesia, or relaxation interventions to help improve patient comfort and tolerability. Some techniques include lidocaine-prilocaine cream (reducing pain related to tenaculum placement), vaginal lidocaine, lidocaine spray, and paracervical block.^2,4^ It is therefore essential to carefully select effective methods according to the profile and specific needs of each patient in order to optimize their comfort during the procedure.

Newer methods include inhaled methoxyflurane (Penthrox), traditionally used in emergency mode, but promising for procedures like hysteroscopy^8^; and transcutaneous electrical nerve stimulation (TENS), a noninvasive technique using high-frequency electrical currents to reduce pain, which is available over-the-counter and inexpensive.^9^ The TENS technique is commonly used by physiotherapists, but is also used during labor and delivery in many countries, such as Spain, Australia, India, Egypt, and Turkey, without side effects.^10,11^ These new approaches could advantageously complement the techniques already in use and offer more options for patients wishing to avoid the significant pain associated with these common gynecological procedures.

Despite these data, there is limited research evaluating the effectiveness of pain management strategies used during these gynecological procedures. This narrative review aimed to summarize the most common pain management techniques during IUD insertion and EMB, and categorize them by their effectiveness. Despite recommendations, uptake of IUDs remains limited in part due to procedural anxiety, particularly among nulliparous and young women.^4,12^ This gap highlights the need for an evidence-based, accessible intervention to improve patient experiences during these procedures.

In summary, in Canada, most guidelines, including those from the SOGC, recommend pre-procedure nonsteroidal anti-inflammatory drugs (NSAIDs) in combination with local lidocaine and anxiety-reducing techniques for pain management during IUD or EMB, particularly for those with high anxiety levels or a history of pain.^3,13^ Other analgesic options, such as Penthrox, may also be suitable in selected cases.^13^

This project aimed to provide pain management options prior to IUD insertions and EMB and to identify and evaluate their efficacy. Ultimately, by empowering both patients and practitioners through education, we aimed to reduce anxiety, enhance satisfaction, and improve the overall quality of women’s health care.

## Methodology

**Figure 1.**
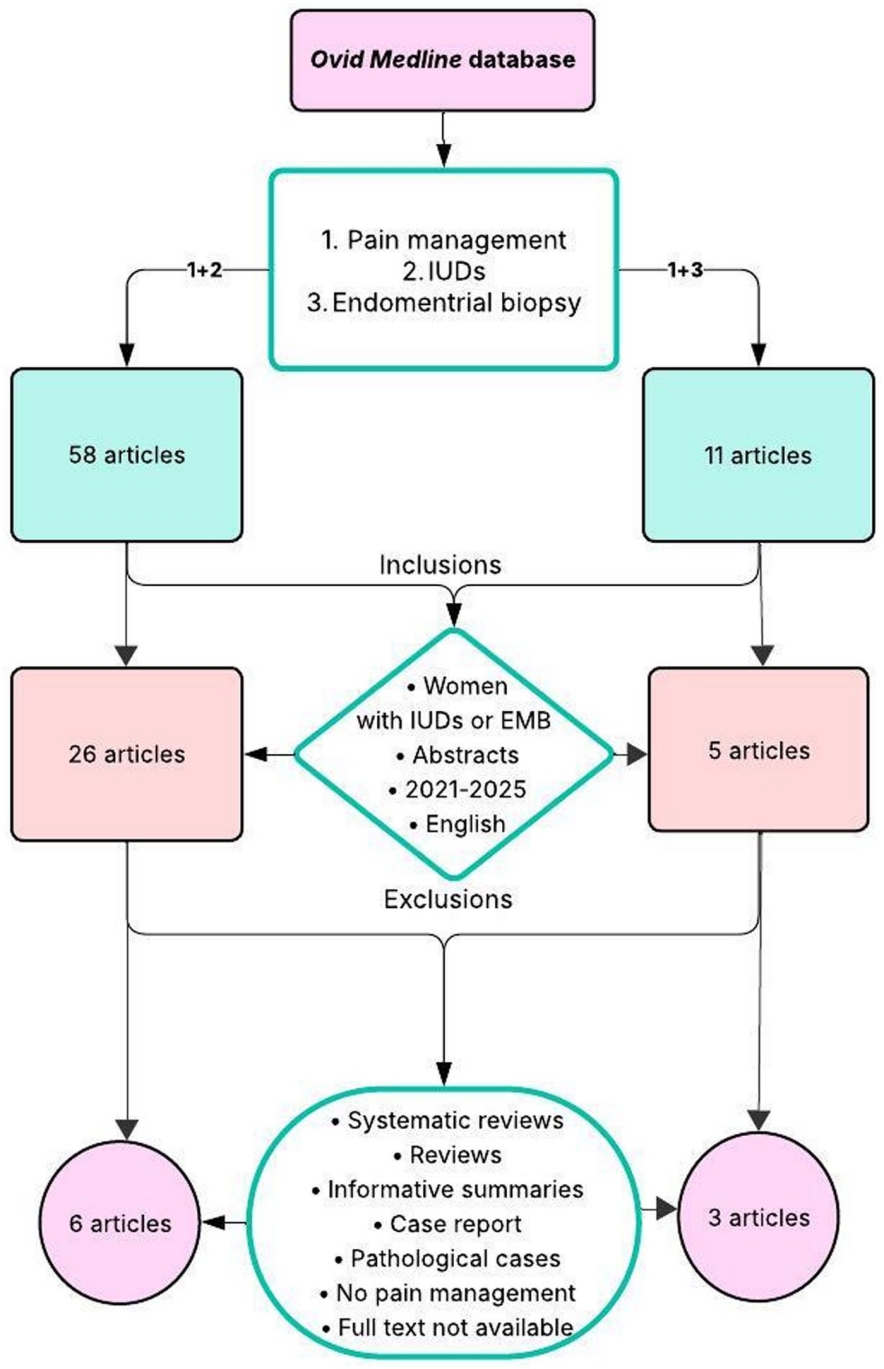
Flowchart used for the “Methodology” section.

This narrative review summarized current evidence on pain management strategies during IUD insertion and EMB. The primary search was used in the *Ovid MEDLINE* database, as it provides comprehensive coverage of medical and clinical literature relevant to this review (Figure 1). PubMed database and the website of the SOGC were used for background reading and for additional references. However, the studies retrieved for synthesis were only selected from Ovid MEDLINE (Table 1). Search combined Medical Subject Headings (MeSH), which included “pain management” combined with “intrauterine devices” and “endometrial biopsy.” Initial searches identified 58 and 11 articles, respectively. The inclusion criteria included a rigorous selection of recent publications from the past 5 years (from 2021 to 2025) to capture recent and clinical evidence and which also reflects current practice standards. Articles involved women undergoing IUD insertion and EMB, assessed pain management strategies, had accessible abstracts, and were also available in English language. The population for this review mainly includes women of childbearing age, as well as women in postpartum and menopausal periods. This selection reduced the number to 26 articles for IUDs and 5 for EMB.

**Table 1.** Studies investigating pain management strategies during IUD insertion or endometrial biopsies in women.

Author	Year	Study design	Sample size	Procedure	Pain management	Study aim	Summary of effectiveness
Wu et al.^9^	2025	RCT	68 English- and Spanish-speaking women in active and 67 in placebo TENS, mean age of 50.9 years in gynecological clinics and hospital-based gynecology clinic in the United States	EMB^a^	TENS device, not excluding NSAIDs, Tylenol, lidocaine, and warm compress	To evaluate whether TENS decreases the pain on a 0–100 with VAS after biopsy, satisfaction and tolerability	Active TENS had 50% score vs 60% in placebo for pain after biopsy (*p* < 0.039) and were overall satisfied at 87.5% with pain control vs 70% in the placebo group (*p* < 0.049).
Ware et al.^14^	2025	Retrospective assessment	28717 Veterans women electronic health records and female at birth receiving care at the VA medical centers (gynecology clinic, women and diverse clinic, primary care clinics or operating rooms), with mean age of 39.5 years in the United States	IUD (authors did not specify which type)^b^	NSAIDs, lidocaine, prostaglandins, opioids, and combinations or other	To assess which pain medication were prescribed, by frequency and how it changed over time	Only 11.4% had a prescribed pain medication. NSAID (ibuprofen) were the most commonly prescribed (8.3%), whereas prostaglandins 1.6%, opioids 0.6% and lidocaine 0.2%. Between 2018 and 2023, pain medication prescription has increased from 10.3% to 13.3%, but remained low compared with that in patients reported pain.
Coskun et al.^15^	2025	RCT	197 women, mean age of 45 years in an outpatient clinic within the department of OBgyn at a hospital in Turkey: 50 women in lidocaine spray; 48 women in dexketoprofen; 50 women in paracervical block and 49 in intrauterine lidocaine	EMB^c^	5 cc of 2% intrauterine lidocaine, oral dexketoprofen (25 mg), cervical lidocaine spray (4 puffs–40 mg) and paracervical block with 2% prilocaine (3 mL) at the 1 and 11 o’clock positions	To measure pain intensity (VAS) during the procedure, right after the procedure and at 30 and 60 min with additional anesthesia	The intrauterine lidocaine group had the lowest pain scores (<5.8/10), whereas the dexketoprofen had the highest one (*p* < 0.001). Paracervical block score was also lower than the lidocaine spray and dexketoprofen group.
Sairally et al.^8^	2024	Prospective observational cohort study	122 women, mean age of 49.1 years in a women’s hospital that is a university teaching hospital in the United Kingdom	Hysteroscopy, EMB and other gynecological procedures^d^	Methoxyflurane (Penthrox), self-administered before the procedure	To assess the acceptability of Penthrox using a Likert scale, the severity of the pain (VAS) and expressing side effects	14% of women with EMB used Penthrox and had a higher pain score of 6.4/10 than the hysteroscopy (4.2/10) or coil change (3.7/10) groups.
Oz and Demirci^16^	2024	RCT	80 women (40 in virtual reality group vs 40 placebo), no age specified in a hospital-based outpatient gynecology clinic	IUD (authors did not specify which type)^e^	VR video of a nature walk with music using virtual reality glasses	To evaluate the effect of virtual reality application with IUD insertion on pain (VAS), anxiety (anxiety scale STAI-S) and patient satisfaction (Likert scale)	The control group showed a higher anxiety score (68.6/100) than the experiment-VR group (23.4/100) with *p* < 0.05. The same applied for the post-procedural pain since they felt less pain with use of video and felt distracted (0.47 v 2.12), and 85% felt satisfied.
Marcelino et al.^17^	2024	RCT	120 women (60 women per group), aged 22–35 within the department of obstetrics and gynecology at a university in Brazil	IUD (levonorgestrel or Copper)^f^	Oral ketorolac (20 mg) 1 h before procedure plus analgesic (1 pill containing dipyrone 300 mg, scopolamine 6.5 µg, hyoscyamine 104 µg, and homatropine 1 mg)	To assess whether ketorolac with analgesic provide effective pain relief during IUD placement (VAS) and to provide an overall pain score	No significant pain score and overall perception of pain during procedure in both groups, although it was significantly lower 10 min post-procedure in the treatment group (2/10) than that in placebo (4/10)
Coleman and Carter^18^	2024	Case series	5 cases aged: 1. 32 2. 40 3. 28 4. 17 5. 22^g^	IUD (authors did not specify which one, although all from same brand)^h^	TENS applications	To assess the rate of pain with the use of TENS after the procedure using the NRS and to compare to prior IUD insertion	Positive patient feedback regarding pain experience using TENS, with NRS <5/10 for all cases. 1. NRS 2/10 2. NRS 1/10 3. NRS 5/10 4. NRS 3/10 5. NRS 5/10
Fowler et al.^19^	2022	Prospective observational study	45 women, aged from 12 to 20 years in clinics and 28 women aged between 12 and 18 with NO analyzed in a hospital-based sedation in the United States	IUD (levonorgestrel)^i^	Ibuprofen or naproxen (500 mg) prior to appointment for both groups. Clinical group received had their iPad, games, or personal phone for distraction and intracervical injection 1% lidocaine 5 mL for all patients. The NO group received nitrous oxide 2 min prior to the procedure	To assess the pain score (VAS) prior, during and 2 min post-procedure	The NO group had significantly lower pain score (18.91/100) than the control group (48.23/100). The NO group was also more likely to recommend the IUD insertion to a friend compared with the control group (*p* < 0.02)
Daykan et al.^20^	2021	RCT	54 nulliparous women, mean age of 25 years in a multicenter outpatient clinic in Israel	IUD (levonorgestrel)^j^	Oral tramadol (50 mg) given pre-procedure or verbal analgesia technique	To assess pain (VAS) with tramadol compared with verbal analgesia (to calm and relax the patient)	No significant pain was observed in the tramadol (4.5/10) group compared with that in the verbal analgesia (4.8/10) group

RCT: randomized controlled trial; EMB: endometrial biopsies; NP: nurse practitioner; PA: physician assistant; PR: physician residents; NSAIDs: nonsteroidal anti-inflammatory drugs; VAS: Visual Analog Scale (0–10 or 0–100; 0 no pain, 10 or 100 very painful); IUDs: intrauterine devices; RN: registered nurse; OBgyns: obstetrician and gynecologist; VR: virtual reality; NRS: Numeric Rating Scale; TENS: transcutaneous electrical nerve stimulation; NO: nitrous oxide.

a Performed by NP, PA, PR, or attending physician.

b Performed by gynecologists, other physicians, NP, RN, PR, and other provider type.

c Performed by a single surgeon, the responsible investigator who has been working at the clinic for 4 years.

d No specification was provided regarding who performed the procedures.

e Performed by OBgyns.

f Performed by a healthcare professional with extensive experience in IUD insertions

g Conduced in-office as outpatients in the United States.

h Performed by the same provider in-office without specifications.

i Performed by pediatric gynecologists.

j Performed by physicians.

After excluding systematic reviews, reviews, pathological cases, summary reviews, studies without pain management intervention, or unavailable full texts, nine relevant studies were selected for further analysis of this review (Table 1). This approach makes it possible to establish recommendations based on the best available evidence, thus ensuring their reliability and applicability in clinical settings.

## Results

The study population across all studies included in this review primarily comprised a diverse range of women, including adolescents, nulliparous women, and parous women, those in perimenopausal, menopausal and postmenopausal stages. The majority of the studies were conducted in outpatient or primary care settings within the department of obstetrics and gynecology of hospitals or universities, and in clinics managed mainly by obstetricians and gynecologists, other physicians, nurses, physician assistants and physician residents where IUDs and EMB are routinely performed. The studies were conducted in the United States, the United Kingdom, Israel, Brazil, and Turkey.

Table 1 includes all nine studies from the “Methodology” section. Each column includes the study design, sample size, procedure, pain management, study aim, and summary of effectiveness of the pain management strategies selected per article.

### Nonsteroidal anti-inflammatory drugs

Most studies describe pain management strategies and their effectiveness during IUD insertion in women. The majority showed that NSAIDs use provided better relief post-procedure with a lower pain score using the Visual Analog Scale (VAS), usually ranging from 0 to 10 or 100 being the most painful. In the study of Ware et al.,^14^ only 11.4% of women had any form of pain management medication prescribed, without clear specification. Ibuprofen was the most common NSAID prescribed with 6.1%, followed by misoprostol 1.6% and hydromorphone 0.1% with other opiates, with an average increase in pain management of 0.52% from 2018 to 2023. However, they did not specify which pain medication provided the most effectiveness. Another study also found a lower pain score post-procedure when using ketorolac combined with another analgesic for IUD insertion with a mean VAS score of 2/10 compared with 4/10 in the placebo group.^17^ However, pain scores during the procedure were similar between both groups, with a mean VAS score of around 8/10 (Table 1).

### Lidocaine

In the study of Coskun et al., 83.7% of women in the intrauterine lidocaine group undergoing EMB had a lower VAS score of less than 5.8/10 during the procedure than those in the other groups (*p* < 0.01).^15^ They also classified a VAS score above 5.8/10 as indicative of severe pain and as per literature. Only 15% of women in the dexketoprofen group had a pain score of less than 5.8/10 in this same study, where indeed they actually had the highest pain score. No significant difference was observed 30 min after the procedure in all groups (*p* = 0.138). None of the results showed atypical hyperplasia, carcinoma, or other malignancies.

### Transcutaneous electrical nerve stimulation

Two studies have used TENS as a pain management strategy and found similar results in which, in fact, it decreases the pain score using the VAS system. Wu et al. found that the women in the TENS group undergoing EMB had a lower pain score with the VAS system and were more overall satisfied by reporting that they would use TENS for a future biopsy (Table 1).^9^ However, the study did not specify how many have also used NSAIDs, Tylenol, lidocaine, antidepressants, benzodiazepines, and opioids prior to the procedure, since they were not excluded from the study from either the active or placebo group. The active TENS group also used a warm compress prior to the procedure. Overall, the active TENS group had a lower VAS score of 50/100 right after the biopsy than the placebo group (60/100). Another case series found that most of the five cases also had a lower pain score (Numeric Rating Scale (NRS)) ranging between 1 and 5 on 10 with the use of TENS during IUD placement, although a nulliparous case had some cramping post-procedure (Table 1).^18^

### Penthrox and nitrous oxide

One study studied the use of Penthrox in gynecological procedures, predominately hysteroscopy, but also others such as EMB.^8^ Their results showed that women with hysteroscopy in office had lower pain score than those in the EMB group (Table 1). However, it was not mentioned whether other analgesics in combination were also used, which could help improve the pain score and patient satisfaction post-procedure. The main side effect was the drowsiness reported by 33% of women. Another study by Fowler et al. found a lower pain score with the use of nitrous oxide (NO) in women undergoing IUD insertion prior to the procedure, but participants were provided other pain relief medication, such as NSAIDs and others mentioned in Table 1.^19^ Participants were also more likely to recommend the use of NO to a friend than the placebo group.

### Virtual reality video

On the other hand, one study used a virtual reality video showing that women undergoing IUD insertion had a lower pain score (VAS) and were also less anxious than the control group.^16^

### Verbal analgesia

Another study also found that verbal analgesia with calming the person, making them feel at ease and telling them that the provider will not cause any harm or pain during the IUD insertion, tends to also have a similar pain score as in the tramadol group.^20^

## Discussion

Pain management during gynecological procedures such as IUD insertions and EMB remains a critical aspect of patient care, influencing both procedural outcomes and patient satisfaction. Numerous studies suggest that healthcare providers recommend NSAID prior to the procedure, but that the pain score is mainly reduced after the procedure. Other common techniques include TENS, virtual videos, and verbal analgesia as described earlier.

Substantial evidence supports the use of various analgesic methods, with Penthrox and inhaled NO emerging as a particularly promising option with reduced pain scores.^8,19^ Overall, the pain relief options that seem to provide the most relief for moderate-to-severe pain in outpatient settings include Penthrox, oral narcotics, and inhaled NO, all of which provide analgesia with minimal sedation. In contrast, IV agents such as NSAID, narcotics, ketamine, propofol, and midazolam provide strong analgesia, but require significant sedation and are more used in emergency or hospital-based procedures.^13^

Ware et al. stated that lidocaine gel, misoprostol, or NSAIDs did not show a significant effect on pain. A more recent article stated that 500–550 mg of naproxen 1 h before the procedure may be beneficial.^14,21^ The researchers even suggested that tramadol had a similar effect in improving pain management during IUD insertion. Another systematic review and metanalysis of randomized control trials in 2019 found that intrauterine anesthetics, anesthetic cervical spray, paracervical block, and oral NSAID provide effective pain control during EMB.^22^ These findings align with the SOGC recommendations, which support continuing these pain management methods within primary care settings.^3^

NO, also known as the *laughing gas*, is commonly used in pediatric outpatient settings, particularly for procedures such as venous cannulation and particularly in anxious or difficult in children. One study reported that difficult children undergoing venipuncture had a mean VAS pain score of 2/10 compared with those in the conventional treatment group with 5/10 (*p* < 0.001), while anxious children or those experiencing pain had a mean VAS score of 1/10 compared with those in the control group with 5/10.^23^ According to the authors, this procedure is relatively easy to perform, but requires an anesthetic delivery system, a suction unit, a scavenging system, and a pulse oximeter, as well as a trained nurse. Another randomized controlled trial (RCT) found that NO administered to nulliparous women (mean age 25 years) undergoing IUD insertion at a reproductive health clinic in New Mexico, United States, resulted in similar VAS pain scores compared with that to the ones receiving oxygen (54 versus 55/100, 2 min post-procedure). However, the NO group reported significantly higher satisfaction with pain management (68% versus 43%, *p* *=* 0.04) using the Likert scale.^24^ Although NO is also used in other clinical settings, such as in outpatient abortion clinics,^25^ it could represent an additional pain management option worth consideration in future SOGC guidelines. Currently, it is not commonly recommended in outpatient or primary care settings, where NSAIDs, opioids, or Penthrox remains the standard option in Canada.^3^

Penthrox, an inhalant that is traditionally used in emergency settings, has demonstrated great potential in outpatient procedures such as hysteroscopy and IUD insertions.^8,26^ This noninvasive inhaled analgesic offers a quick onset of action and is self-administered with the supervision of the healthcare provider, with more than 80% of women accepting it as a pain management analgesic during gynecological procedures, reflecting its strong potential for gynecological care. In Canadian primary care and gynecological settings, the use of Penthrox is becoming significant.^13^ Its use has been recently introduced in Canadian primary care settings after following its use in Australia, the United Kingdom, and some parts of Europe. Most IUD insertion and EMB are performed in community-based or primary care clinics where access to intravenous or complex anesthesia is limited and most providers will use local or minimal anesthesia. Current evidence suggests that Penthrox may be a feasible and accessible option for use in office-based procedures, potentially offering value in outpatient settings, especially in rural or remote areas where hospital-based sedation is limited.

Moreover, noninvasive techniques, such as TENS, widely used by physiotherapists and during labor and delivery, present a multidisciplinary approach that aligns with current best practices in pain management and as a valuable adjunct during gynecological procedures.^9–11^ Although TENS has not been routinely adopted in primary care or during outpatient gynecological settings, its ease of use, safety profile, and cost-effectiveness make it an attractive option for clinicians managing procedures such as IUD insertion and EMB. Moreover, it provides a non-pharmacological, easily administered option with the combination of analgesics. Their devices are also portable and require minimal training, which will allow family physicians and other healthcare providers to integrate them into their practice. This approach is particularly beneficial for patients with contraindications to systemic NSAIDs, such as gastrointestinal and kidney impairment or intolerance to opioids.^27,28^

Another new study also reported that adolescents and adults face certain barriers to IUDs.^29^ Examples include providers’ knowledge of IUDs placement and gaps in insurance coverage, and some remain uncomfortable counseling about contraception methods. More importantly, adolescents and adults may have anxiety pre-procedure, which, in turn, may predict a higher pain score during the procedure itself. Other factors include patients being worried due to social media videos about IUDs. Overall, their study emphasized building trust between patient and provider relationships for IUD placement.^29^ Therefore, it is essential to have healthcare providers who are skilled in these gynecological procedures and who can help patients feel more at ease and comfortable by reducing anxiety.^21^

The study by Hutchison and Espey suggested collecting patient prior history that can impact IUD insertion pain such as previous gynecological history with pain, intimate partner or sexual violence, anxiety, depression, and high levels of anticipated pain.^30^

In most articles, nulliparous women tend to have a higher score of pain than parous women with C-section or vaginal birth. This is most likely explained by cervical stenosis and no passage of childbirth.^5,20,31^ These data suggest reinforcing pain management strategies to help reduce the pain in nulliparous women in an outpatient setting by decreasing their anxiety and increasing their satisfaction.

The integration of education on pain management strategies, counseling about the procedure itself, its potential discomfort and available pain relief methods can further enhance these outcomes. Studies have shown that women who receive clear explanations and reassurance experiences can lower their anxiety and also report less procedural pain.^4,12,20,21,30^ This combination empowers women, leading them to feel more in control and less anxious during gynecological procedures. This trend should encourage healthcare professionals in primary care to adopt a more comprehensive methodology tailored to individual needs and preferences, which, in turn, enhances their overall satisfaction and compliance.

By clearly describing and explaining pain management options to our patients and by understanding their concerns and their previous history, we can foster a sense of confidence and preparation among patients, ensuring they are informed and less anxious regarding their gynecological procedures. Following this review, we recommend conducting larger, well-designed RCTs, as well as prospective cohort and observational studies, to compare and evaluate different multimodal analgesic approaches. Overall, these types of studies would help optimize pain management strategies, assess safety and feasibility in diverse clinical contexts, and enhance patient satisfaction in outpatient and primary care settings during gynecological procedures.

## Limitations

This review has some limitations. First, there was variability in the pain rating scales used across the studies selected for this review. Some employed the VAS ranging from 0 to 10 or 0 to 100, whereas others used the NRS or the Likert-type scales assessing pain intensity and satisfaction of the participants. This diversity of scales may have limited the comparability of pain outcomes and influenced the perceived effectiveness of analgesic use. Although the TENS technique seemed to show consistency in reduced scores in both studies used for this review, direct comparison with modalities such as Penthrox or others is limited, as the use of pre-procedure of analgesics was not specified in some studies. Moreover, the type of study, sample size, geographic location, and pain management techniques may have also created some bias and affected both pain outcomes, patient anxiety, and satisfaction. In some cases, if the procedure was performed by different practitioners, such as physicians, nurses, or other healthcare providers, who have different levels of experience and expertise, it may also have influenced participants’ pain outcomes or perceptions. Finally, the absence of standardized pre procedures analgesic use across the studies may have further influenced pain outcomes reported by the participants.

## Conclusion

In conclusion, this narrative review highlights the integration of current pain management strategies available during gynecological procedures such as IUD insertion and EMB. The evidence suggests that a multimodal approach combining a pharmacological and non-pharmacological approach can improve patient pain levels and satisfaction, with particularly the use of inhaled methoxyflurane (Penthrox), NO, TENS, and verbal analgesia in combination with NSAIDs in routine women’s health care. Future research should focus on larger RCTs, cohort, and observational studies to further evaluate the effectiveness, safety, and feasibility of these pain management strategies.

By improving provider awareness and patient education, clinicians can help reduce anxiety, enhance satisfaction, and promote broader acceptance of essential gynecological procedures in outpatient clinics and in primary care settings. Implementing standardized pain management protocols and training healthcare providers as needed could further optimize patient satisfaction and procedural outcomes.
